# Is vaccine the magic bullet for malaria elimination? A reality check

**DOI:** 10.1186/1475-2875-9-S3-S1

**Published:** 2010-12-13

**Authors:** Roma Chilengi, Jesse Gitaka

**Affiliations:** 1 KEMRI-Wellcome Trust Research Programme, P.O. BOX 230, Kilifi, Kenya; 2 University of Oxford, Nuffield Department of Health, Centre for Clinical Vaccinology and Tropical Medicine, UK

## Abstract

Malaria remains a major health burden especially for the developing countries. Despite concerted efforts at using the current control tools, such as bed nets, anti malarial drugs and vector control measures, the disease is accountable for close to a million deaths annually. Vaccines have been proposed as a necessary addition to the armamentarium that could work towards elimination and eventual eradication of malaria in view of their historical significance in combating infectious diseases. However, because malaria vaccines would work differently depending on the targeted parasite stage, this review addresses the potential impact various malaria vaccine types could have on transmission. Further, because of the wide variation in the epidemiology of malaria across the endemic regions, this paper proposes that the ideal approach to malaria control ought to be tailor-made depending on the specific context. Finally, it suggests that although it is highly desirable to anticipate and aim for malaria elimination and eventual eradication, many affected regions should prioritize reduction of mortality and morbidity before aspiring for elimination.

## Background

Malaria transmission is falling in some parts of Africa as anti-malarials, bed nets and other vector control measures become more widely available [1-4]. However, malaria disease continues to be a major public health disaster with persistent transmission in vast areas and it is clear that additional control measures are required. Indeed epidemiological data indicates that malaria is still a global health priority and the statistics of estimated 5 billion people exposed and close to 1 million deaths year remain valid [5-7]. This is especially important in the vast parts of sub-Saharan Africa where the social and ecological environments render current control tools blunt. Recent successes in malaria control using other approaches highlight the need and the potential impact that could be gained from an efficacious vaccine [8-10]. Historically, vaccination has proved to be one of the most effective approaches to controlling infectious diseases and many authorities believe that it will not be possible to move from control to elimination without the addition of a malaria vaccine to the armamentarium [9]. However, despite concerted international efforts, an efficacious vaccine against malaria remains elusive.

The leading malaria vaccine candidate in development RTS,S, which is currently undergoing phase III field evaluation in African children, has progressed based on demonstrated efficacy against clinical malaria recently reported to be around 50% in the field [11]. This good news of a possible vaccine against malaria in the foreseeable future has revived the possibility of enhanced malaria control and perhaps incited the call for malaria elimination and eventual eradication. However, it is important to examine current aspirations for malaria elimination in the light of key historical experiences and scientific facts.

**Figure 1:**
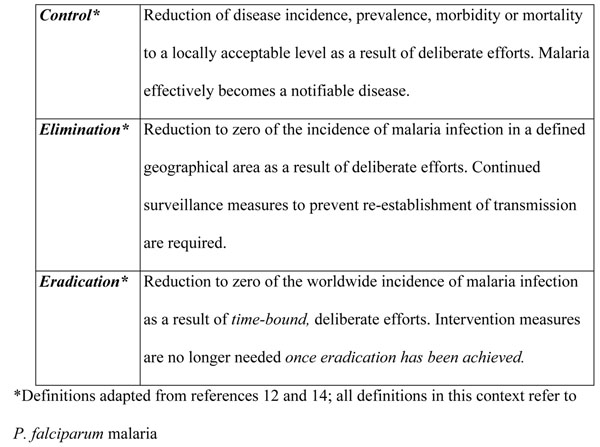
Definitions of key terminology.

Following the Eighth World Health Assembly resolution on transition from malaria control to its eradication, interruption of malaria transmission was achieved in many countries of the temperate belt, and mortality from the disease decreased dramatically. By 1970, about 1 billion people were freed from the risk of malaria, but it had already become clear during the 1960’s that the available methods of control would not interrupt malaria transmission in tropical Africa [12-14]. The point of note here is that expectations from control measures ought to be based on the realistic possibility of what available tools can achieve. Figure 1 describes the relevant key terminology applicable to malaria elimination.

## How would a vaccine against malaria affect transmission?

Transmission can be expressed in terms of simplified mathematical models based on easy to visualize parameters, the most useful of which is the Macdonald model [15]. This model describes the concept of basic case reproduction rate (R_0_), which, in short, is the number of secondary cases arising from a single case in a fully susceptible human population. It is a tool for understanding and a way of thinking, but it cannot be accurately applied in field situations [16]. Based on the possible transmission dynamic factors in the Macdonald formula, this review looks at the potential impact of vaccines targeting the various malaria parasite stages would have on the basic case reproduction rates.

**Figure 2:**
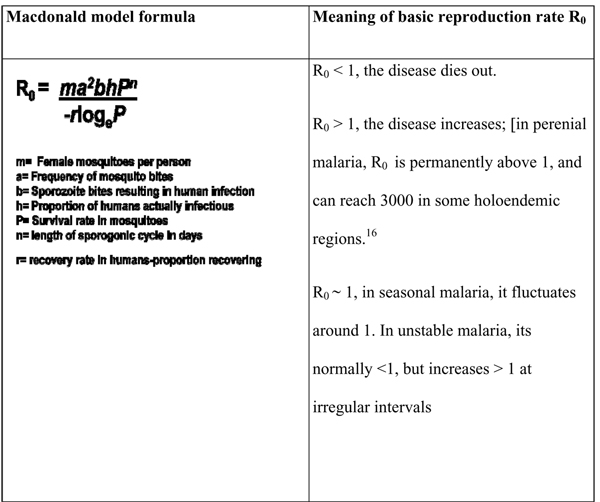
The Macdonald mathematical formula.

Figure 2 shows the Macdonald mathematical formula and the summarized meaning of the basic reproduction rate R_0._

### Pre-erythrocytic vaccines

Ideally, pre-erythrocytic vaccines would induce some form of sterile protection that prevents infection by the sporozoite beyond the liver stage [1,17-19]. However, a review of the global malaria vaccine pipeline [20] shows that all the current candidate vaccines have a profile aiming at “partial protection” against malaria episodes meaning that at best, they would not completely interrupt the malaria parasite cycle in all vaccinees. The irradiated sporozoite vaccine approach is thus far the only one that has shown to be highly efficacious at protection of humans as well as animals, but this protection is yet to be demonstrated in malaria endemic populations [21,22]. The most advanced candidate vaccine RTS,S recently showed an adjusted efficacy against clinical episodes of malaria at 53% (95% CI 28-69; P<0.001) in Kenyan and Tanzanian children [11].

Such pre-erythrocytic stage vaccines would impact transmission by reducing both “**b**” and “**h**” and thus overall, reduce R_0_ by a proportion that could possibly correlate to its protective efficacy. It is difficult at this stage to assume that such an impact will roll on to either affect the length of the sporogonic cycle or survival rate in mosquitoes. What is apparent however, is that other control measures, such as ITNs [1,23-25], use of repellants [26,27], indoor residual sprays [28,29] and indeed prophylactic anti-malarial drug usage [30-32], would be synergistic to pre-erythrocytic vaccine effects. In the Macdonald model, these control methods would contribute to reductions in R_0_ by additionally pulling both “**a**” and “**m**” lower and ultimately shrinking “**h**” which respectively, are the frequency of mosquito bites, female mosquitoes per person and proportion of humans actually infectious.

### Blood stage vaccines

According to the WHO’s malaria vaccine Rainbow tables, there are currently at least 15 different sub-unit candidate vaccines targeting the parasite asexual (blood) stages in clinical development [20]. There are several hypothesized mechanisms through which asexual stage vaccines may function: that antibodies bind parasite antigens to sufficiently agglutinate and prevent release of merozoites, or block invasion of erythrocytes leading to protection against clinical disease and/or its severity [33-36]; that vaccines such as MSP3 and GLURP would induce cytophilic classes of antibodies killing parasites with help from monocytes [37-39]; and that others (such as PfEMP1, Rifins, Pf332) would enhance splenic clearance or complement mediated lysis, or diminish parasite nutrition and growth or reverse endothelial adherence and glycoprotein binding to result in prevention of toxic effects [8].

In essence, these vaccines are expected to prevent manifestation, or limit the severity of clinical malaria disease in immunized individuals, when they get infected. This was well illustrated in the results of the trial of malaria vaccine Combination B in Papua New Guinea, which demonstrated a 62% (95% CI 13-84) reduction in parasite density in children but no effect on infection and in fact, a higher incidence of morbid episodes associated with the variant parasites (with FC27-type) not covered in the vaccine [39]. One major foreseeable challenge for vaccine candidates targeting the blood is strain-specificity of the vaccines antigens and the extent to which they would cover parasite polymorphisms encountered in field coupled with the variability displayed in the cell invasion pathways *P. falciparum*[40-43].

On the transmission front however, we can expect infections to continue within a vaccinated population for several transmission cycles. The variables **m, a, b**, and **P** in the Macdonald’s formula would be unaffected (at least during the first encounter following deployment of the vaccine). The proportion of humans actually infectious “**h**” would be reduced while the recovery rate “**r**” would greatly increase. All factors remaining the same, it is plausible that “**b**”, the sporozoite bites resulting in human infection, would be reduced by the next generation of parasites and eventually reduction in “**b”** would substantially contribute lowering R_0_ within the vaccinated population. The standard control measures of early diagnosis and effective chemotherapy would greatly enhance this impact [30-32,44,45]. Current recommendations to use two or more blood schizonticidal drugs with independent modes of action and different biochemical targets aims at both improving the efficacy and retarding the development of resistance to the individual components of the combination. This concept has been realized in multiple-drug therapy for leprosy, tuberculosis and cancer and, more recently in antiretroviral treatments. In malaria, this has also been the approach with the development of such drugs as sulphadoxine-pyrimethamine, atovaquone-proguanil, mefloquine-sulphadoxine-pyrimethamine and lately artemisinin-based combinations. In the context of reducing malaria transmission, drugs that are implicated in gametocytogenesis, such as sulphadoxine/pyrimethamine [46] may actually enhance transmission by causing an increase in “**h**”; the proportion of humans actually infectious.

### Sexual stage vaccines

Vaccines targeting the sexual stages of the parasite are termed transmission-interrupting vaccines because they would stimulate antibodies that inhibit exflagellation and fertilization of gametocytes, that render them non-infectious for the mosquitoes when taken up during a blood meal [47]. Antibodies could also block the process by which ookinetes develop into oocysts and prevent transmission of infectious sporozoites to humans [47-49]. Examples of potential vaccine antigens like this include Pfs25, Pfs48/45 and Pfs230. Currently, there is no candidate vaccine targeting this stage that has made it to clinical field evaluation, but there are two candidates in pre-clinical evaluation, both of which are based on Pfs 25 [20].

The hallmark of this category of vaccines is that they would have no immediate clinical benefit to recipients in terms of protection against malaria infection and disease, but will benefit the wider community [1,49]. In terms of transmission model dynamics, if transmission-blocking vaccines are effectively and completely deployed in a population viewed as a homogeneous compartment, they would disrupt transmission by shrinking “**P**”, the survival rate in mosquitoes. By the next generation of parasites, the proportion of humans actually having the infection “**h**” as well as the survival rate in mosquitoes “**P**” would be remarkably lowered. The key challenge to vaccine development here is that entire populations would have to be immunized and the vaccine effects should last through several transmission cycles. However, other vector control measures including ITN use, in-door residual spraying, use of repellents as well as adverse climatic conditions against the mosquito vector (such as drought) would greatly enhance these effects as earlier discussed.

## Gauging the expectations

**Figure 3:**
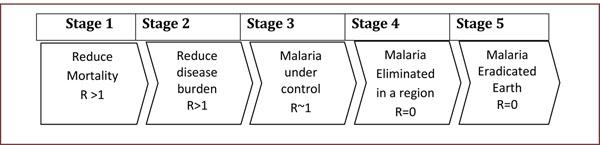
Expression of stages of malaria control towards eradication.

From an epidemiological view point, progress towards malaria elimination can be viewed in terms of reduction in disease specific attributable mortality; reductions in the overall disease burden; the extent to which a disease under control; proportion to elimination of the disease; and then eradication and ultimately extinction as shown in Figure 3.

Aspirations for malaria elimination and eventual eradication should indeed be the vision or ultimate goal of any malaria control programme. However, while grappling with high case fatality rates, overwhelming disease burden and failure to implement or sustain available control measures, it may be too optimistic, if not unrealistic to consider elimination issues in many contexts in Africa. Figure 3 illustrates in a simplified way, the stages at which malaria endemic regions may be placed depending on the level of control, or the lack of it that the region experiences.

The progress of endemic countries or regions on this scale is affected by many factors including, but not limited to; its level of endemicity by entomological inoculation rates, efficiency at implementation of available tools, social economic situation of the region, health systems efficiency, climatic conditions as well as political stability including that of neighbouring regions. Therefore, the immediate or short to medium term goals of a particular region should depend on what stage they are in these series.

## So is elimination all wishful thinking?

Interventions using current tools can result in major reductions in malaria transmission and the associated disease burden; however, in high transmission settings they are insufficient to drive prevalence below the pre-elimination threshold [50]. A malaria vaccine offers, therefore, great potential for improved malaria control, particularly in Africa, where effective mosquito control over long periods has proved difficult or impossible to maintain [51]. Indeed recent successes in malaria control using other approaches highlight the need and the potential gains that could come from an efficacious vaccine [8,10]. However, to adequately measure vaccine impact will require enhanced surveillance and standardized reporting mechanisms. Unfortunately, the large range of R_0_ estimates in literature confirms the fact that malaria control presents variable challenges across its transmission spectrum and a ‘‘one-size-fits-all’’ malaria control strategy would be inefficient in the broader context of malaria elimination [16]. Large reductions in transmission from targeted control are possible only if programmes are able to identify those who are bitten most, and specific interventions packaged and implemented efficiently. Vector control measures can impact reductions to the 1% parasite prevalence threshold in low- to moderate-transmission settings especially when the main vectors are primarily endophilic (indoor-resting), provided a comprehensive and sustained intervention programme is efficiently managed. In high-transmission settings and areas, where vectors are mainly exophilic (outdoor-resting), additional new tools that target exophagic (outdoor-biting), exophilic, and partly zoophagic mosquitoes will be required [50,52,53]. Depending on which stage (see Figure 3) in control a particular region might be at, specific tailor-made interventions would be required to move from one stage to the next. In areas where R_0_ is low such as those around stage 3, local elimination of malaria may be practical and concerted efforts should focus on that goal [52]. The immediate realistic focus of control should be reducing the mortality and disease burden in general, for areas where R_0_ may be high such as those around stage 1 and 2.

On the other hand, the impact of the malaria interventions should not be limited to the estimated level of efficacy and coverage alone. The potential impact of vaccines could generally be wider than expected due to synergies and doubling of effects, which is hard to theoretically predict. For example, RTS,S was observed to have an impressive reduction in severe disease incidence in Mozambican children despite being a pre-erythrocytic stage vaccine [54]. This trial was designed to primarily assess vaccine efficacy against clinical malaria disease, which at six months was found be 29·9% (95% CI 11·0–44·8; p 0·004) while efficacy against severe malaria was 57·7% (95% CI 16·2–80·6; p 0·019). Could synergies between a partially protective malaria vaccine and currently available control tools further enhance the impact of vaccine?

## What a malaria vaccine will not do

As the discussion on the possibility of malaria elimination in some African contexts goes on, it is imperative that expectations from the potential role of an efficacious vaccine are moderated. To do so, it is important to consider what not to expect from the current generation of vaccines in clinical development:

*1. Be 100% protective efficacy:* None of the vaccines currently in clinical development will be close to 100% protective efficacy. The malaria vaccine Technology Roadmap rightly predicts that by 2015, the licensed vaccine could achieve a 50% reduction in malaria deaths and severe illness among young children in sub-Saharan Africa and by 2020, license a vaccine that can achieve an 80% reduction [55].

*2. Be deployed by the vaccine developer:* Once an efficacious vaccine is licensed, it will be available to governments and their Ministries of Health to include in their control programmes, procure and deploy. African countries who have had to change their national anti-malarial drug policy will recognize the complexities of harmonizing various national treatment guidelines, developing effective in-service training, ensuring adequate drug supply and educating the patient population [56,57].

*3. Eliminate malaria from countries in stages 1 & 2:* Existing tools may be sufficient to reduce the burden of disease and bring it under control in many low transmission areas. However, the situation in much of sub-Saharan Africa is such that partially protective vaccines currently in clinical development, may not in themselves bridge the control gap [1,9].

*4. Count the numbers to document control and elimination:* The capacity to accurately account for the impact of malaria vaccines towards elimination will be critical. Improved surveillance and reporting systems will be necessary to demonstrate any vaccine impact .

*5. Reduce the cost of malaria control:* Even if the vaccine will ultimately be paid for by donors, sub-Saharan African governments should expect successful deployment of a vaccine to come at a cost to their already strained health budgets. Thus, availability of an efficacious (and affordable) malaria vaccine should be viewed initially as a cost before the rewards of control will be realized.

It is notable that currently, there is a significant effort to categorize diseases by their global morbidity and mortality impact and this has developed substantially during the last decade, epitomized by the reporting the Global Burden of Diseases and the Disease Control Priorities project [57,58]. Unfortunately, despite these efforts, the evidence base for allocating resources for malaria control on a global scale is still poor [57-59] and no meaningful improvement will come without affected regions themselves taking on serious initiatives and responsibility.

## Need to visit the drawing board

Although there is renewed motivation towards malaria elimination, most of the vaccines currently in the global portfolio were not conceived in the context of malaria elimination [43]. The focus on vaccines that are deployable through the expanded programme on immunization is certainly useful in targeting the most vulnerable and most affected by the disease, but may not at all deal with the reservoir hosts available in older children and semi immune adults. It does also appear evident that sub-unit vaccines with a single parasite antigen target may not be sufficient to interrupt malaria transmission especially in areas with higher than moderate transmission. To attain to malaria elimination, research and development must continue even when partially protective vaccines become available. More efforts on combined and multi-stage vaccines with potent adjuvants will be necessary ingredients for the malaria elimination agenda [51].

This call for malaria elimination should be extended to all stakeholders from funders of malaria vaccine work through endemic country governments, research institutions, control programmes and down to the individual family faced with the daily challenge of malaria sickness. There is need for an entire paradigm shift if malaria elimination is to eventually be realized and it is not enough to simply pump more funds into research and development [57,58]. The mere possibility of having a vaccine against malaria highlights the fact that there is now a need, more than ever before, to rethink how to integrate all available tools and resources towards malaria elimination.

## Conclusion

Is vaccine the magic bullet for malaria elimination? Probably not today. The global community working towards malaria control and eventual elimination is faced with many difficult challenges which cannot be fixed by a magic bullet. The fact that malaria transmission and clinical manifestation is so varied demands a variety of approaches be made in the fight against the scourge. Strategic planning for malaria control should consider R_0_, the spatial scale of transmission and human population density in tailor-making interventions so that multiple, integrated and sustained control methods are focused in populations where R_0_ is highest [16].

The current efforts for vaccine development have rightly targeted *falciparum* malaria, which is the major cause of morbidity and fatality. However, because Plasmodium inter-species characteristics are said to be products of evolutionary dynamics, it is important to be circumspect and not forget the possibility of a “new malaria problem” (more so with *Plasmodium vivax* ), once *Plasmodium falciparum* is eliminated [62].

The protracted fight against malaria should have taught us that the parasite is a resilient enemy able to mount various escape strategies and therefore, it must be approached with multi pronged approaches. Malaria vaccines currently in clinical development represent pre-clinical knowledge and thinking of 10-20 years ago. The present landscape however, demonstrates the need to design vaccines with the goal of eliminating and eventually eradicating malaria.

## Competing interests

The authors declare that they have no competing interests.
